# Canopy rainfall partitioning and hydrological nitrogen and phosphorus losses under contrasting fertilization regimes in a flue-cured tobacco field

**DOI:** 10.3389/fpls.2026.1939056

**Published:** 2026-10-13

**Authors:** Yu Chen, Wenyin Zi, Shan Yang, Yunxue Li, Xiangdong Xu, Jing Shi, Jianjun Chen

**Affiliations:** 1 College of Resources and Environment, Yunnan Agricultural University, Kunming, China; 2 Key Laboratory for Improving Quality and Productivity of Arable Land of Yunnan Province, College of Resources and Environment, Yunnan Agricultural University, Kunming, China; 3 College of Agriculture and Horticultural Technology, Yunnan Vocational College of Agriculture, Kunming, Yunnan, China

**Keywords:** canopy rainfall partitioning, flue-cured tobacco, hydrological nutrient export, organic fertilizer substitution, soil aggregate stability

## Abstract

**Introduction:**

Fertilization can alter both soil properties and crop canopy characteristics, with potential implications for hydrological nutrient export, yet field evidence on the relationship between canopy rainfall redistribution and nutrient-loss patterns remains limited.

**Methods:**

During the 2023 tobacco-growing season, we conducted a field experiment in a flue-cured tobacco–faba bean rotation system in the Erhai Lake Basin, China. We compared conventional fertilization (CF), organic substitution (OS, in which chemical-fertilizer N under CF was fully replaced on an equal-N basis with commercial organic fertilizer), and optimized fertilization (OF, with chemical-fertilizer N and P inputs reduced by 25% and K input increased by 25% relative to CF). Soil properties, aggregate stability, tobacco growth and nutrient status, canopy rainfall partitioning during three late-season rainfall events, and seasonal N and P losses through runoff and leaching were quantified.

**Results:**

Compared with CF, OS reduced soil bulk density by 8.53% and increased soil organic matter, porosity, and moisture content by 9.71%, 11.61%, and 15.35%, respectively, while also improving aggregate stability. OS increased leaf N concentration, maximum leaf area, and yield by 25.44%, 17.25%, and 11.14%, respectively, despite nonsignificant differences in leaf area index and canopy coverage. Fertilization regime significantly affected canopy interception and throughfall, with OS showing the numerically highest interception and lowest throughfall across the three events. OS reduced surface runoff by 7.04%, runoff-mediated TN and TP losses by 22.46% and 20.63%, and leaching-mediated TN and TP losses by 24.70% and 30.61%, respectively. OF showed more moderate soil and plant responses but lowerrunoff-mediated TP and leaching-mediated TN and NH_4_^+^-N losses. Exploratory analyses indicated numerically stronger associations of canopy interception with runoff-mediated than with leaching-mediated nutrient losses.

**Discussion:**

Contrasting fertilization regimes were associated with coordinated soil, plant, canopy-hydrological, and nutrient-export responses. Rainfall partitioning differed despite similar *LAI* and canopy cover, indicating that bulk canopy-size metrics do not fully capture canopy hydrological functioning. The stronger association of canopy interception with runoff-mediated losses suggests a closer relationship between canopy rainfall redistribution and surface nutrient transport, providing a process-oriented basis for evaluating nutrient-loss mitigation.

## Graphical Abstract

**Figure:**
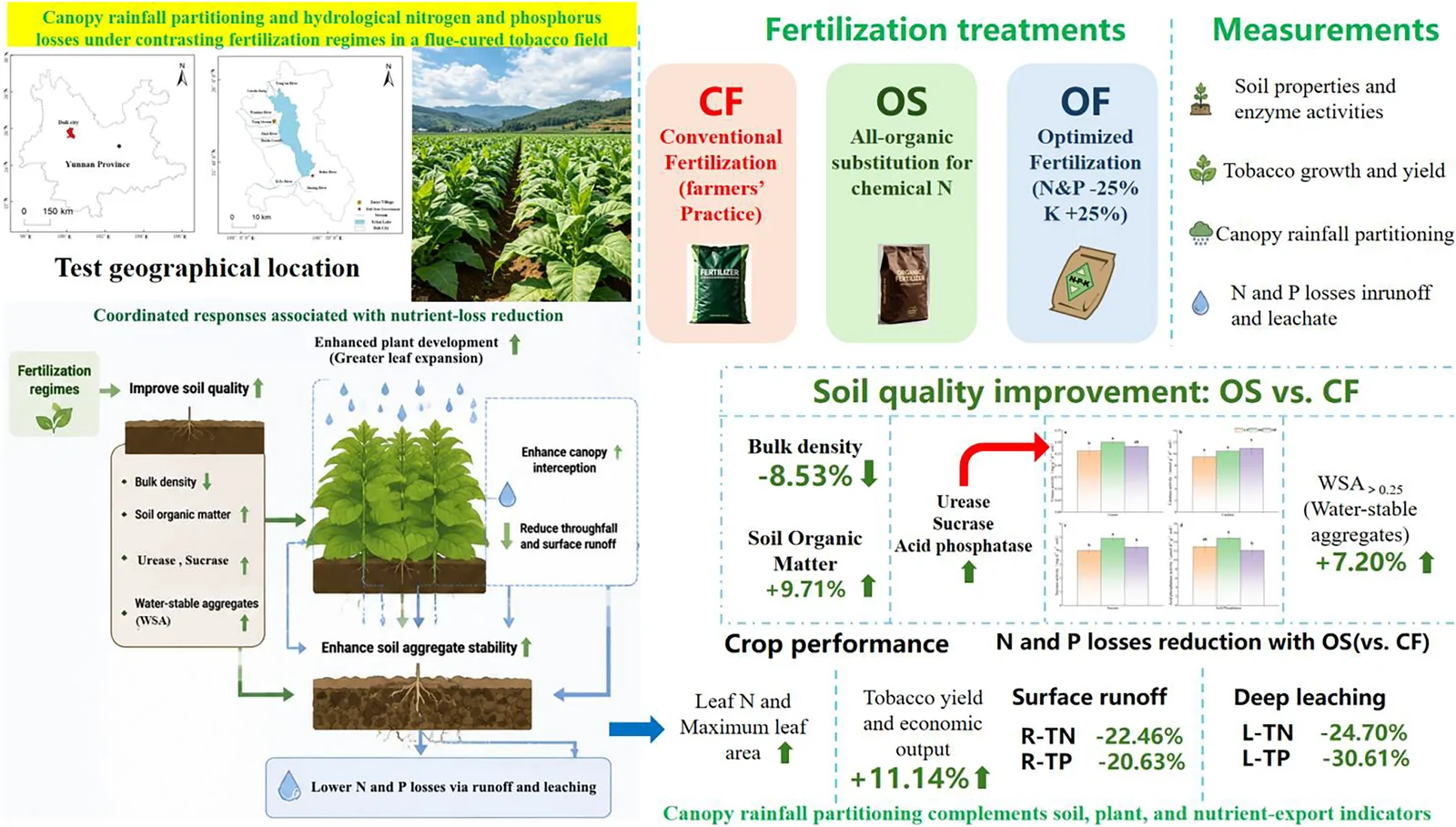

## 1 Introduction

Efficient nutrient management is essential for balancing crop growth, nutrient acquisition, and agricultural sustainability. Nitrogen (N) and phosphorus (P) are essential nutrients that support leaf development, biomass accumulation, and yield formation. However, when nutrient inputs exceed crop uptake demand, surplus nutrients can accumulate in the soil and subsequently be transported from agricultural fields through surface runoff and deep leaching (Li et al., 2022; Gong et al., 2022; Zhou W. et al., 2022; Wang et al., 2024). Therefore, the effects of fertilization on nutrient losses extend beyond direct changes in the soil nutrient pool and involve interconnected soil-plant-hydrological processes. On the one hand, fertilization directly modifies soil nutrient availability, nutrient retention, and water-holding capacity (Das et al., 2023). On the other hand, by modifying plant nutritional status, leaf development, and aboveground canopy formation, fertilization may indirectly alter rainfall partitioning before precipitation reaches the soil surface (Zheng et al., 2018). In intensive agricultural systems where periods of rapid crop growth coincide with concentrated rainfall, elucidating the plant-soil-hydrological coupling induced by nutrient management is critical for understanding the mechanisms governing nutrient transport from farmland.

The crop canopy represents the first biological interface between atmospheric precipitation and the soil surface. Rainfall is partitioned by leaves and stems into interception, throughfall, and stemflow (Fernandes et al., 2021; Petit et al., 2022; Saidin et al., 2022), thereby altering both the amount and spatial distribution of water delivered to the soil. These processes can influence runoff generation, infiltration, soil-particle detachment, and nutrient transport. Canopy rainfall partitioning is jointly controlled by rainfall characteristics and plant structural and functional properties (Dong et al., 2020). Therefore, fertilization-associated changes in plant development may influence how rainfall is intercepted, retained, and redistributed before reaching the soil surface, with potential consequences for subsequent water and nutrient transport (Yang et al., 2024). However, compared with the well-established effects of fertilization on soil fertility, crop productivity, and nutrient losses, the hydrological functioning of crop canopies under contrasting fertilization regimes remains poorly understood. In particular, it remains unclear whether fertilization-associated changes in plant development and canopy characteristics are accompanied by measurable differences in rainfall partitioning, and whether these hydrological responses are subsequently associated with nutrient export through surface runoff and subsurface leaching.

This knowledge gap is particularly relevant in agricultural systems within plateau lake basins, where seasonal rainstorms, intensive fertilizer inputs, and crop growth periods frequently coincide. The Erhai Lake Basin is a typical intensively farmed watershed on the Yunnan Plateau. More than 85% of its annual precipitation occurs between May and October, making the crop-growing season a high-risk period for nitrogen and phosphorus transport from farmland. Flue-cured tobacco is an economically important crop in the region, with a cultivated area exceeding 3,500 ha (He et al., 2023), and its vigorous vegetative growth stage overlaps substantially with the regional rainy season (Bai et al., 2018; Wang et al., 2022; Zhong et al., 2022; Ouyang et al., 2024). This seasonal concurrence creates a hydrologically sensitive observation period during which crop development, canopy rainfall partitioning, and nutrient transport can be observed simultaneously. Although a single growing season cannot capture interannual variability, synchronized measurements during this period provide valuable field evidence for characterizing their co-occurrence and coordinated responses under contrasting fertilization regimes. Previous studies have shown that replacing chemical fertilizers with organic fertilizers can improve soil organic matter content, aggregate stability, nutrient retention, and crop productivity (Gong et al., 2022; Zhou W. et al., 2022; Wang et al., 2024). Meanwhile, hydrological studies in agricultural systems have demonstrated that rainfall characteristics strongly determine the occurrence and intensity of runoff and leaching, whereas crop canopies can substantially modify rainfall distribution before water reaches the soil surface (Fernandes et al., 2021; Petit et al., 2022; Saidin et al., 2022; Tamagno et al., 2022; Ouyang et al., 2024). However, these responses have generally been examined separately, and their joint variation under contrasting fertilization regimes remains insufficiently characterized. In particular, limited field evidence is available on whether differences in canopy rainfall partitioning can occur when conventional canopy-size metrics such as leaf area index (*LAI*) and canopy cover remain similar. Such a pattern would indicate that bulk canopy-size metrics alone are insufficient to describe canopy hydrological behavior. It also remains unclear whether canopy rainfall partitioning is more closely associated with surface nutrient export than with subsurface leaching. Therefore, simultaneous assessment of soil conditions, plant responses, canopy hydrology, and nutrient transport is needed to characterize their coordinated variation without assuming a predefined causal sequence.

To address these questions, we conducted an *in situ* field experiment during the 2023 flue-cured tobacco-growing season in the Erhai Lake Basin. We compared conventional fertilization (CF), organic substitution (OS, in which the chemical fertilizer N supplied under CF was fully replaced on an equal N basis by commercial organic fertilizer), and optimized fertilization (OF, with chemical-fertilizer N and P inputs reduced by 25% and K input increased by 25% relative to CF). Because these treatments differed in fertilizer composition and nutrient stoichiometry, they were evaluated as complete fertilization regimes rather than as an isolated test of fertilizer source. Soil physicochemical properties and aggregate stability, tobacco growth and nutrient status, canopy rainfall partitioning, and N and P export through surface runoff and subsurface leaching were quantified within the same field experiment. Specifically, we asked whether (1) contrasting fertilization regimes were accompanied by distinct soil, plant, and nutrient-export responses; (2) canopy rainfall partitioning differed among fertilization regimes even when leaf area index (*LAI*) and canopy cover showed no significant treatment differences; and (3) canopy rainfall redistribution and soil structural properties exhibited different strengths of exploratory association with surface and subsurface nutrient export. Accordingly, this study aimed to characterize the co-occurring soil, plant, canopy-hydrological, and nutrient-export responses under contrasting fertilization regimes and to explore associations among these components, rather than to test a predefined trait mediated causal pathway. By examining these responses concurrently within a common field setting, this study provides a basis for identifying coordinated soil–plant–hydrological response patterns and offers field-relevant information for evaluating fertilization strategies that balance tobacco production with the reduction of hydrological N and P losses in rainfall-sensitive agricultural systems.

## 2 Materials and methods

### 2.1 Site description

The field experiment was established and conducted from 2022 to 2024 in Zuoyi Village, Xizhou Town, Dali City, Yunnan Province (25°51′N, 100°08′E), This study focused on the 2023 flue-cured tobacco-growing season, for which synchronized measurements of canopy rainfall partitioning, plot-scale runoff, and leaching were available to support the integrated soil-plant-canopy-hydrological analysis. The experimental area is adjacent to Dali Agricultural Comprehensive Experimental Station of the Environmental Protection Research and Monitoring Institute of the Ministry of Agriculture and Rural Affairs. It is located in the core planting area of high-quality flue-cured tobacco in Yunnan Plateau. The typical flue-cured tobacco-faba bean dryland rotation mode is adopted in the field. The altitude of the test area is about 1980 m, which belongs to the northern subtropical plateau monsoon climate. The annual temperature difference is small, the frost-free period is up to 285 days, and the annual average sunshine hours are 2345 h. During the experiment, the local average daily temperature was 14.8 °C, and the average annual precipitation was 1048 mm. The precipitation distribution was uneven during the year, and more than 85% was concentrated in the rainy season of May-October. The soil type tested in the experiment was paddy soil. The basic physical and chemical properties of the 0–20 cm surface soil before the start of the experiment are detailed in **Table 1**.

**Table 1:** Soil physical and chemical properties before experiment.

Rotation pattern	pH	SOM (g·kg^-1^)	S-TN (g·kg^-1^)	S-TP (g·kg^-1^)	TK (g·kg^-1^)	NH_4_^+^ (mg·kg^-1^)	NO_3_^-^ (mg·kg^-1^)	AP (mg·kg^-1^)
Flue-cured tobacco-Faba bean	7.07 ± 0.15	64.91 ± 3.02	2.35 ± 0.30	1.12 ± 0.37	34.12 ± 3.62	19.07 ± 3.00	25.37 ± 2.51	29.53 ± 2.96

The data are mean ± standard deviation. SOM, means Soil Organic Matter; S-TN, means Soil Total Nitrogen; S-TP, means Soil Total Phosphorus; TK, means Total Potassium; NH_4_^+^, means Ammonium Nitrogen; NO_3_^-^, means Nitrate Nitrogen; AP, means Available Phosphorus.

### 2.2 Experimental design and field management

Three fertilization treatments were established in this experiment, with three independent replicate plots for each treatment, resulting in a total of nine experimental plots. Each plot constituted an independent experimental unit. The tobacco cultivar Yunyan 87 was used across all plots. Basal fertilizer was applied on 15 April; seedlings were transplanted on 27 April; and two top-dressing events were conducted on 29 May and 6 June, respectively. Harvesting was completed on 15 September. Fertilizer application rates for each treatment are summarized in **Supplementary Table S1**. The treatments were as follows: (1) Conventional fertilization (CF), representing the standard fertilization practice commonly adopted by local farmers; (2) Organic substitution (OS), in which synthetic fertilizer-derived N under CF was completely replaced by an equivalent amount of N supplied by commercial organic fertilizer, while maintaining the same total N input as CF; and (3) Optimized fertilization (OF), in which synthetic fertilizer-derived N and P inputs were each reduced by 25%, whereas synthetic fertilizer-derived K input was increased by 25% relative to CF. Because fertilizer source, organic-C input, and N:P:K supply were not manipulated independently, the experimental design does not permit attribution of treatment responses to any individual fertilizer component. Accordingly, all treatment effects were interpreted at the level of the complete fertilization regime. In all treatments, solid organic fertilizer was applied solely as basal fertilizer. The first top-dressing consisted of liquid organic fertilizer (300 kg·ha^−1^), compound fertilizer, and potassium sulfate; the second top-dressing comprised liquid organic fertilizer only (300 kg·ha^−1^). Each plot measured 24 m² (6 m × 4 m), arranged in ridge cultivation: ridge height = 0.20 m, ridge width = 0.50 m, inter-ridge spacing = 0.60 m, intra-ridge plant spacing = 0.50 m, with five ridges per plot and eight plants per ridge-yielding 40 plants per plot. Plots were physically isolated using soil embankments. A drainage outlet (0.15 m in height) was installed at the lower end of each plot to channel surface runoff into a dedicated collection tank (3.0 m × 1.0 m × 0.6 m; total volume = 1.8 m³), the top of which was sealed with corrugated steel plates to exclude precipitation. A schematic illustration of the experimental plot layout is provided in **Figure 1a**. Additionally, a subsurface leachate collection system was installed 30–60 cm beneath each plot. It consisted of a collection box (40 cm × 25 cm × 30 cm) with a 2.5 cm bottom aperture, layered with nylon mesh and 5 cm fine quartz sand, then backfilled with native soil. The outlet was connected to a buried PVC collection pipe (150 cm long, 11 cm diameter), allowing leachate to drain by gravity. The entire assembly was buried with the box base at 60 cm depth; the pipe extended 30 cm aboveground and was sealed to prevent contamination (**Figure 1c**).

**Figure 1:**
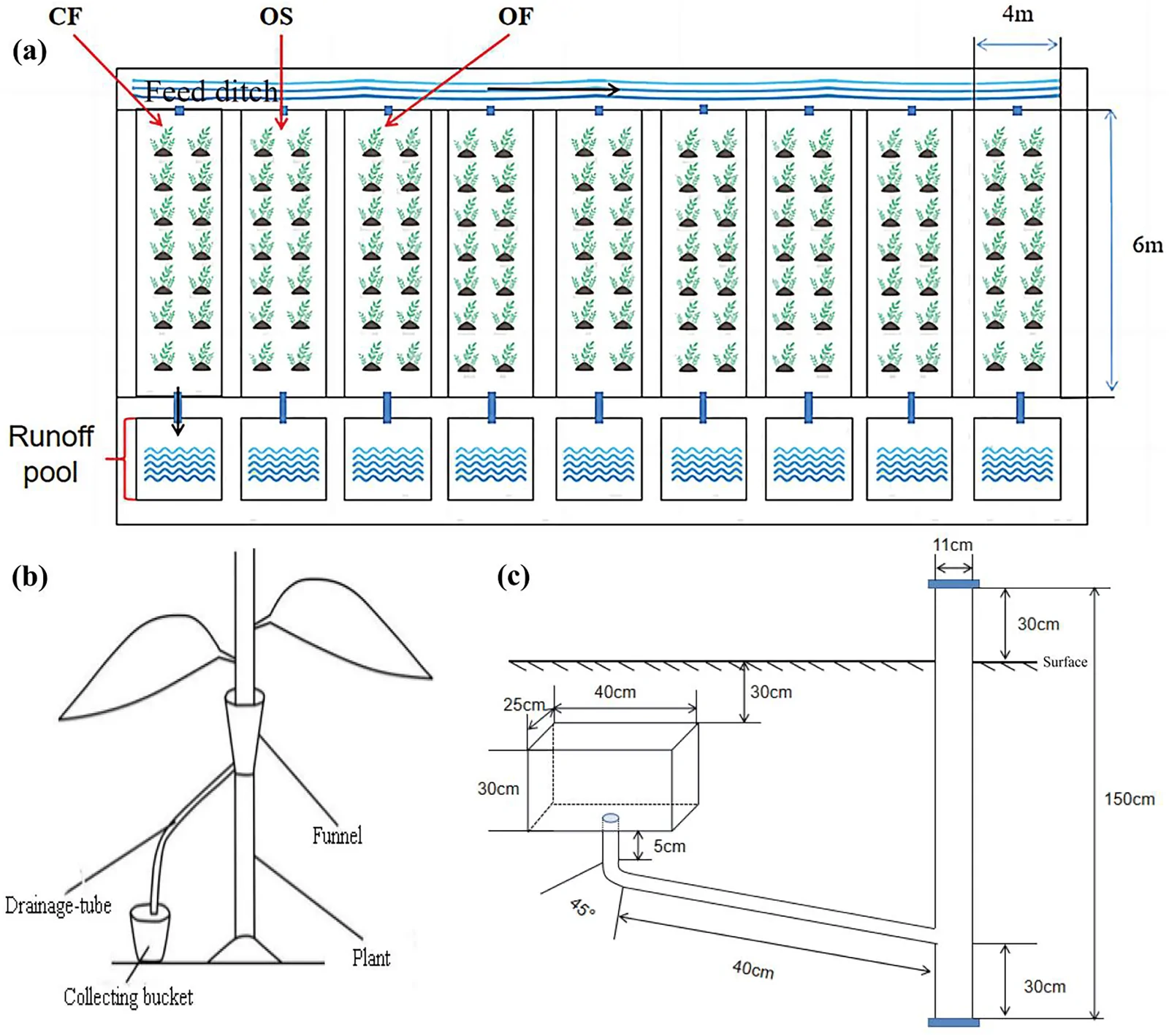
Schematic representation of the experimental plots and hydrological monitoring systems. **(a)** Layout of the surface-runoff monitoring plot; **(b)** Modified stemflow collection deviceby Lyu et al (Lü et al., 2020); and **(c)** subsurface leachate collection system.

### 2.3 Measurement of canopy rainfall partitioning

At the flowering and topping stage, three representative non-border plants were randomly selected from the central rows of each plot and permanently tagged. Gross rainfall for each monitored event was measured using a standard manual rain gauge installed in an unobstructed open area adjacent to the experimental plots (Because other meteorological characteristics were not continuously monitored, between-event differences were interpreted as integrated rainfall-event effects rather than being attributed solely to rainfall amount). Throughfall was collected using four cylindrical collectors (10-cm opening diameter; 78.54 cm^2^ collection area) positioned around each monitored plant, with one collector in each cardinal direction. Collector positions were stratified within a 0–25 cm radial zone from the main stem, with two collectors randomly positioned within 0-12.5 cm and two within >12.5–25 cm; the assignment of radial strata to directions was randomized among plants. All collectors remained fixed throughout the monitoring period. After each rainfall event, collected water volumes were measured and converted to equivalent water depths based on collector area. The mean of the four collectors was used as the plant-level throughfall estimate, and the mean of the three monitored plants was used as the plot-level plant-centered throughfall estimate. Stemflow was collected from the same plants using spiral collars fitted around the basal stems and connected by tubing to sealed collection containers. The collected volume was measured after each rainfall event and converted to equivalent water depth based on the mean ground area occupied by an individual plant (**Figure 1b**).

### 2.4 Sample collection

1. Soil sampling: Before the application of fertilization treatments, five soil cores were randomly collected from the 0–20 cm layer of the experimental field and mixed to form a composite sample for the analysis of initial soil properties. After the tobacco harvest, soil samples from the 0–20 cm plow layer were collected from each plot using the same five-point composite method. Each composite sample was then divided into two sub-samples by quartering: one for the determination of routine soil chemical properties, and the other for the analysis of soil aggregate size distribution and stability.
2. Rainfall collection: A standard manual rain gauge was installed in an open area adjacent to the experimental plots to measure gross rainfall amount. Rainfall samples for chemical analysis were collected using a separate, clean polyethylene bottle equipped with a funnel, which was deployed only during rainfall events.
3. Runoff water sampling: After each runoff-producing rainfall event, the water depth in each runoff collection pool was measured to calculate the total runoff volume. A mixed runoff water sample was collected by agitating the pool water thoroughly and then filling a 500 mL polyethylene bottle. To prevent overflow during continuous rainfall, an additional sample was collected if the water level reached 80% of the pool’s capacity. Samples were immediately transported to the laboratory, filtered as required for different analyses, and stored at 4 °C until analysis, which was performed within 7 days. After sampling, the pools were cleaned and emptied in preparation for the next event.
4. Leachate water sampling: During non-fertilization periods, leachate was sampled at approximately 30-day intervals, with additional sampling conducted on the day following each fertilization event. At each sampling time, the entire volume of leachate accumulated since the previous sampling was completely withdrawn and measured. The collected leachate was thoroughly homogenized, and a representative 500-mL subsample was transferred into a polyethylene bottle for subsequent chemical analysis. After sampling, the PVC collection pipe was completely emptied to initiate the next accumulation interval. Samples were immediately transported to the laboratory, stored at 4 °C, and analyzed within 48 h.
5. Crop agronomic traits and yield: At the flowering and topping stage, three representative plants were randomly selected from each experimental plot, permanently tagged, and equipped with stemflow collection devices. At physiological maturity, plant height, stem girth, and the length and width of the largest functional leaf were measured on the tagged plants according to the national standard YC/T 142–2010. During harvest, fresh leaf biomass was recorded, and leaf area was estimated using a standardized allometric method. Fresh leaves and stems were then heated at 105 °C for 30 min and subsequently dried at 75 °C to constant weight to determine dry matter content and plant dry biomass. The dried leaf samples were retained for the determination of total N, P, and K concentrations, as described below.

### 2.5 Sample analyses

1. Soil analysis: Soil bulk density (BD) was measured using the core method. Total soil porosity was calculated as Porosity=(1−BD/PD)×100, where PD represents soil particle density**;** Soil aggregate-size distribution was determined by dry sieving, whereas water-stable aggregates were fractionated by wet sieving into >2, 0.25-2, and <0.25 mm size classes following established aggregate fractionation procedures with modifications (Yoder, 1936; ElIiott, 1986). Aggregate stability was characterized using the proportion of water-stable aggregates >0.25 mm (WSA_>0.25_), mean weight diameter (MWD), and geometric mean diameter (GMD). Soil gravimetric moisture content was determined by oven-drying fresh soil at 105 °C to constant mass (Weber et al., 2023); Soil pH was measured potentiometrically in a 1:2.5 soil-to-deionized-water suspension; Soil electrical conductivity (EC) was measured in a 1:5 (w/v) soil-to-deionized-water extract after shaking for 1 h (He et al., 2012). Soil cation exchange capacity (CEC) was determined using the 1 M ammonium acetate (NH_4_OAc) method buffered at pH 7 following established procedures (Kabala and Jedrzejewski, 2024). Soil organic matter (SOM) and soil organic carbon (SOC) were determined by externally heated potassium dichromate oxidation following the method of Mebius (1960). Dissolved organic carbon (DOC) was extracted from fresh soil with deionized water at a soil-to-water ratio of 1:5 (w/v) by shaking for 1 h at 20°C, followed by centrifugation and filtration through a 0.45 μm membrane; Particulate organic carbon (POC) and mineral-associated organic carbon (MAOC) were separated by physical size fractionation modified (Cambardella and Elliott, 1992). Briefly, air-dried soil (<2 mm) was dispersed in 0.5% (w/v) sodium hexametaphosphate at a soil-to-solution ratio of 1:5 and shaken for 18 h. The suspension was then passed through a 53 μm sieve; material retained on the sieve (>53 μm) was designated as the particulate organic matter fraction, whereas material passing through the sieve (<53 μm) was designated as the mineral-associated organic matter fraction. Organic C concentrations in both fractions were determined by potassium dichromate oxidation, and POC and MAOC contents were calculated on an equivalent bulk-soil mass basis; Total Nitrogen (S-TN) was determined using the semi-micro Kjeldahl method; Soil ammonium nitrogen (NH_4_^+^) was measured using KCL leaching-indophenol blue colorimetric method; Soil nitrate nitrogen (NO_3_^-^) was determined using the phenol disulfonic acid colorimetric method; TP (S-TP) was measured using NaOH melting-molybdenum antimony anti-colorimetric method; AP was extracted with 0.5 mol·L^−1^ NaHCO_3_, and the extract was determined by Mo-Sb colorimetric method; TK was measured using NaOH melting-flame photometric method (Bao, 2000). The urease activity was determined by the sodium phenol-sodium hypochlorite colorimetric method. catalase activity was determined by potassium permanganate titration. Sucrase activity was determined by 3,5-dinitrosalicylic acid colorimetry. Acid phosphatase activity was determined using the p-nitrophenyl phosphate (pNPP) colorimetric method (Xing et al., 2022).
2. Water analysis: Water samples were thoroughly homogenized immediately prior to subsampling. Unfiltered aliquots were used for the determination of TN (alkaline potassium persulfate digestion ultraviolet spectrophotometry) and TP (potassium persulfate oxidation spectrophotometry). Another sample was filtered by 0.45 μm filter membrane, and then NH_4_^+^-N, NO_3_^--^N and TDP (Continuous flow injection apparatus method) were determined.
3. Plant nutrient analysis: Dried leaf samples from the three tagged plants within each plot were pooled to form one composite sample at the plot level and ground to a fine powder. The ground plant material was digested with H_2_SO_4_–H_2_O_2_. Leaf total N concentration was determined using the micro-Kjeldahl method, total P concentration using the molybdenum–antimony colorimetric method, and total K concentration using flame photometry.

### 2.6 Data processing and analysis

(1) Leaf area index (*LAI*) was calculated as follows (Shao et al., 2017):


$$\text{Leaf area index}(LAI)=\frac{k_{1}\rho \sum_{i=1}^{m}\sum_{j=1}^{n}L_{ij}\times W_{ij}}{\alpha }$$

Where *k_1_*= 0.6345 (tobacco leaf area correction coefficient); *ρ* = the planting density (1.67 plants·m^-2^); *Lij* and *Wij* are the maximum length and width of the j-th leaf of the i-th tobacco plant; *α* is the number of measured plants.

(2) Canopy cover (*Cc*) was determined using (Li et al., 2016; Cai et al., 2020):


$$Canopy\;cover\;(Cc)=1-e^{-e\lambda_{0}k_{2}LAI}\;$$

In the formula: *λ_0_k_2 =_*0.71. LAI means Leaf area index.

(3) Canopy interception (mm)= Rainfall (mm) - Stemflow (mm) - Throughfall (mm).

(4) Canopy interception rate (%) = (Canopy interception/Rainfall) × 100;

The stemflow rate of a single crop (%)= stemflow volume of the crop/Rainfall×100 (Ouyang et al., 2024). (Throughfall rate were calculated analogously).

(5) The total amount of nitrogen lost from surface runoff is equal to the sum of the product of pollutant concentration and volume in each runoff water in the whole monitoring cycle (a complete anniversary). The calculation formula is as follows:


$$P=\frac{\sum_{i=1}^{n}C_{i}\times V_{i}}{2400}$$

Of which: *P*——Pollutant loss, kg·ha^-1^ ;

*C_i_*——Concentration of nitrogen in the ith runoff water, mg·L^-1^;

*V_i_*——Volume of the ith runoff water, L.

(6) The cumulative leaching loss of nutrient X was calculated as follows:


$$L_{x}=0.1\sum_{i=1}^{n}C_{x,i}\times V_{i}$$

where *L_x_* is the cumulative leaching loss of nutrient X during the monitoring period; *C_x,i_* is the concentration of nutrient X in the leachate collected during the i-th sampling interval; *V_i_* is the total leachate volume collected during that interval; and *n* is the total number of sampling intervals.

(7) The percentage of water-stable aggregates >0.25 mm (WSA_>0.25_, %) was calculated as:


$${WSA}_{>0.25}=\frac{M_{>0.25}}{M_{a}}$$

Where *M*_>0.25_ = the mass of aggregates >0.25 mm; $M_{a}$ = total soil sample mass.

(8) The Mean weight diameter (MWD, mm) was calculated as:


$$MWD=\frac{\sum_{i-1}^{n}(\bar{X_{i}}W_{i})}{\sum_{i-1}^{n}Wi}$$

Where $\bar{X_{i}}$ = mean diameter of the *i*-th aggregate fraction (mm); $W_{i}$ = mass proportion of the *i*-th fraction.

(9) The Geometric mean diameter (GMD, mm) was calculated as:


$$GMD=\exp\frac{\sum W_{i}ln\bar{X_{i}}}{\sum W_{i}}$$

(10) Statistical analysis: Raw data were organized using Microsoft Excel 2010, and statistical analyses and graphical visualization were performed using SPSS version 21.0, R version 4.5.2, and OriginPro version 2021. Statistical significance was determined at *P* < 0.05. Treatment effects on variables measured once at the plot level were evaluated using one-way analysis of variance (ANOVA), followed by Fisher’s least significant difference (LSD) test when the overall treatment effect was significant. Repeatedly measured variables, including canopy rainfall partitioning and event-specific runoff-mediated nutrient losses, were analyzed using linear mixed-effects models, with fertilization treatment, rainfall event, and their interaction as fixed effects and plot identity as a random intercept. Pairwise comparisons of estimated marginal means were adjusted using the Holm method. Event-scale relationships between canopy interception and runoff- and leaching-mediated TN and TP losses were examined using linear regression, with R² and corresponding P values reported. Pearson correlation analysis was used to assess pairwise associations among soil aggregate stability, canopy interception, hydrological transport, and nutrient-loss variables, whereas Mantel tests were used to evaluate multivariate associations between soil physicochemical properties and grouped hydrological responses representing water-loss volume, runoff-mediated nutrient loss, and leaching-mediated nutrient loss. Linear mixed-effects models and estimated marginal mean comparisons were conducted in R version 4.5.2 using the lme4, lmerTest, and emmeans packages. Different lowercase letters indicate significant pairwise differences among treatments at *P* < 0.05.

## 3 Results

### 3.1 Effects of fertilization regimes on soil physicochemical properties and enzyme activities

#### 3.1.1 Soil physicochemical properties

Soil physicochemical properties differed among fertilization regimes (**Table 2** and **Supplementary Table S2**). Relative to CF, OS significantly reduced soil bulk density by 8.53% and increased soil moisture content and porosity by 15.35% and 11.61%, respectively(*P* < 0.05). OS also increased EC and CEC by 20.00% and 11.83%, respectively, together with increases of 9.71%, 8.09%, and 26.74% in SOM, SOC, and DOC. In addition, TK, NH_4_^+^-N, and AP were significantly higher under OS than under CF by 20.69%, 22.54%, and 11.69%, respectively (*P* < 0.05). OF produced more moderate changes, reducing bulk density by 3.10% and increasing soil porosity, CEC, and TK by 5.97%, 6.21%, and 11.02%, respectively, whereas AP decreased by 7.37% relative to CF. Compared with OF, OS had significantly higher soil moisture content, porosity, EC, CEC, DOC, S-TP, TK, NH_4_^+^-N, and AP. Soil pH, S-TN, and NO_3_^-^ did not differ significantly among treatments.

**Table 2:** The changes of physical and chemical properties of soil under different treatments.

Treatment	CF	OS	OF
pH value	7.15 ± 0.07 a	7.15 ± 0.17 a	7.05 ± 0.23 a
Bulk density (g·cm^-3^)	1.29 ± 0.02 a	1.18 ± 0.02 c	1.25 ± 0.02 b
Moisture content (%)	19.48 ± 0.57 b	22.47 ± 0.62 a	20.47 ± 0.07 b
Soil porosity (%)	48.22 ± 0.84 c	53.82 ± 0.45 a	51.10 ± 0.85 b
Electrical Conductivity (mS·cm^-1^)	0.35 ± 0.02 b	0.42 ± 0.02 a	0.37 ± 0.02 b
Cation Exchange Capacity (cmol·kg^-1^)	13.53 ± 0.25 c	15.13 ± 0.31 a	14.37 ± 0.31 b
SOM (g·kg^-1^)	67.74 ± 1.93 b	74.32 ± 3.01 a	70.63 ± 3.01ab
SOC (g·kg^-1^)	39.29 ± 1.12 b	43.11 ± 1.75 a	40.96 ± 1.75 ab
DOC (g·kg^-1^)	0.86 ± 0.04 b	1.09 ± 0.05 a	0.96 ± 0.08 b
S-TN (g·kg^-1^)	2.02 ± 0.19 a	2.42 ± 0.29 a	2.27 ± 0.16 a
S-TP (g·kg^-1^)	1.00 ± 0.06 ab	1.15 ± 0.09 a	0.94 ± 0.09 b
TK (g·kg^-1^)	33.40 ± 1.83 c	40.31 ± 0.82 a	37.08 ± 0.61 b
NH_4_^+^ (mg·kg^-1^)	7.41 ± 0.44 b	9.08 ± 0.47 a	8.10 ± 0.46 b
NO_3_^-^ (mg·kg^-1^)	18.09 ± 1.19 a	20.94 ± 1.51 a	19.65 ± 1.41 a
AP (mg·kg^-1^)	78.43 ± 1.44 b	87.60 ± 3.95 a	72.65 ± 2.43 c

EC, is Electrical Conductivity; CEC, is Cation Exchange Capacity; SOM, is Soil Organic Matter; SOC, is Soil Organic Carbon; DOC, is Dissolved Organic Carbon; S-TN, is Soil Total Nitrogen; S-TP, is Soil Total Phosphorus; TK, is Total Potassium; NH_4_^+^, is ammonium nitrogen; NO_3_^-^, is nitrate nitrogen; AP, is available phosphorus. The data are mean ± standard deviation. Different letters in the same row indicated that the parameter was significantly different between different treatments, using one-way analysis of variance, *P* < 0.05.

#### 3.1.2 Soil enzyme activities

Fertilization regime significantly affected urease, sucrase, and acid phosphatase activities but had no significant effect on catalase activity (**Figure 2**). Compared with CF, OS increased urease and sucrase activities by 14.47% and 22.45%, respectively (*P* < 0.05). Relative to OF, OS increased sucrase and acid phosphatase activities by 15.42% and 22.49%, respectively (*P* < 0.05).

**Figure 2:**
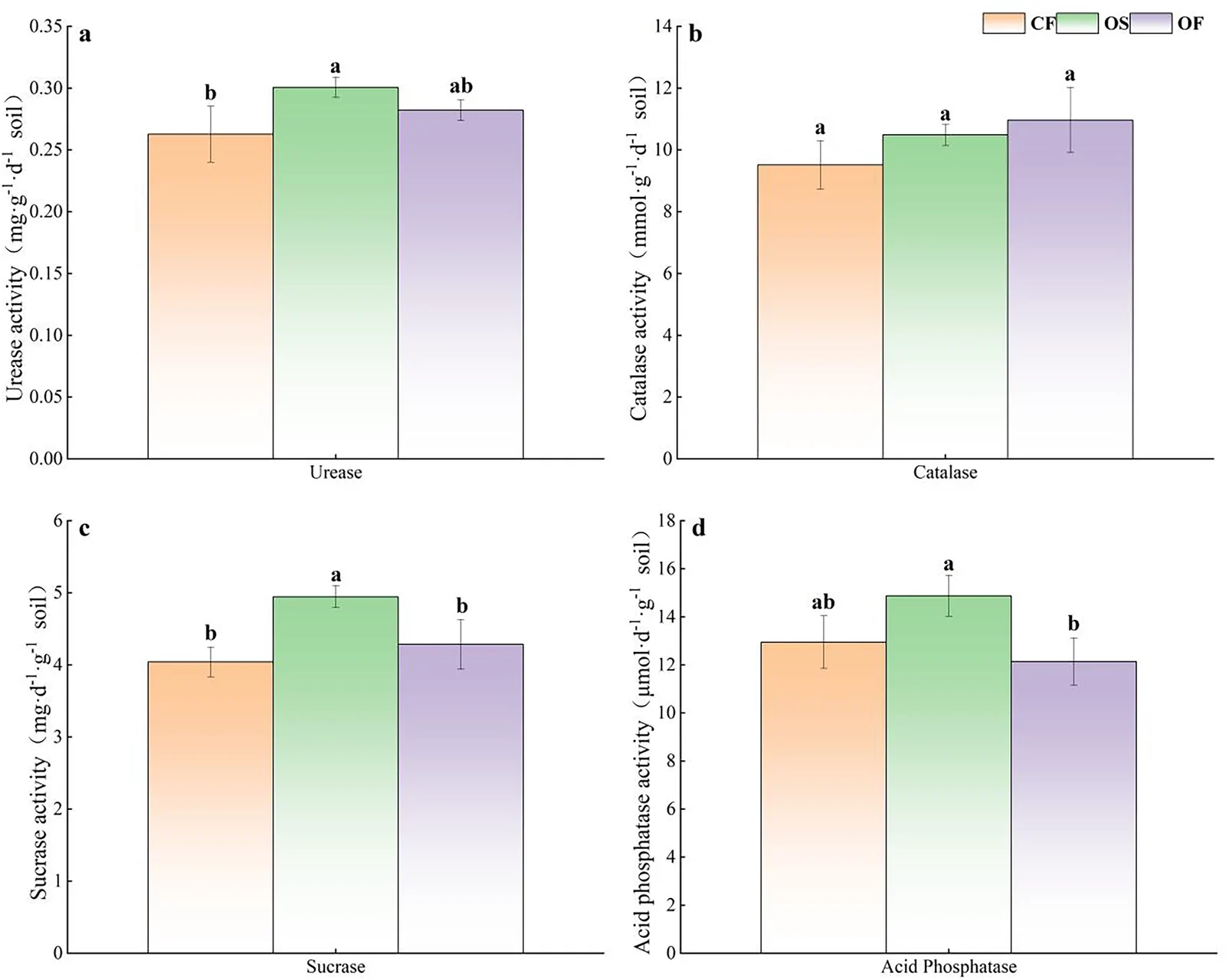
Soil enzyme activity under different fertilization treatments. **(a)** The activity of Urease under different fertilization treatments; **(b)** the activity of Catalase under different treatments; **(c)** the activity of Sucrase under different treatments; **(d)** the activity of Acid Phosphatase under different treatments. All data are mean ± SD, the error bars indicate standard deviation (n=3) and letters express significant differences (*P* < 0.05).

### 3.2 Effects of fertilization regimes on tobacco nutrient status, leaf expansion, canopy traits, and yield

Fertilization regime significantly affected leaf expansion, leaf N accumulation, plant survival, and productivity, whereas plant height, stem girth, maximum leaf length, and leaf P and K concentrations did not differ significantly among treatments (**Table 3**; *P* > 0.05). Relative to CF, OS increased the number of available leaves, maximum leaf width, maximum leaf area, and leaf total N concentration by 21.20%, 8.86%, 17.25%, and 25.44%, respectively (*P* < 0.05). These responses were accompanied by a higher survival rate (94.17% vs. 86.67%) and increases of 8.79%, 11.14%, and 11.14% in dry matter yield, tobacco yield, and economic output value, respectively. OF generally showed intermediate numerical values for maximum leaf area, tobacco yield, and economic output, whereas its survival rate (90.83%) was also significantly higher than that under CF but did not differ significantly from OS. Neither *LAI* nor *Cc* differed significantly among fertilization regimes (**Figure 3**; *P* > 0.05), despite the significant treatment responses observed for several individual-leaf traits and plant survival.

**Table 3:** The effects of different fertilization treatments on agronomic traits and yield of flue-cured tobacco.

Treatment	CF	OS	OF
Plant height (cm)	94.17 ± 8.97 a	99.11 ± 7.98 a	93.33 ± 7.72 a
Stem girth (cm)	10.33 ± 1.15 a	10.69 ± 1.14 a	10.13 ± 0.73 a
Number of available leaves per plant (piece)	14.67 ± 2.45 b	17.78 ± 2.82 a	15.11 ± 2.15 b
Maximum leaf length (cm)	58.28 ± 4.85 a	62.54 ± 6.64 a	59.41 ± 5.85 a
Maximum leaf width (cm)	21.56 ± 1.04 b	23.47 ± 1.52 a	21.8 ± 1.52 b
Maximum leaf area (cm^2^)	798.57 ± 94.11 b	936.31 ± 156.42 a	824.98 ± 125.23 ab
TN content of tobacco leaves (%)	1.69 ± 0.02 b	2.12 ± 0.07 a	1.81 ± 0.11 b
TP content of tobacco leaves (%)	0.60 ± 0.04 a	0.66 ± 0.09 a	0.69 ± 0.06 a
TK content of tobacco leaves (%)	2.24 ± 0.11 a	2.17 ± 0.02 a	2.36 ± 0.15 a
Survival rate (%)	86.67 ± 2.89 b	94.17 ± 1.44 a	90.83 ± 1.44 a
Dry matter yield (kg·ha^-1^)	487.05 ± 9.61 b	529.88 ± 17.34 a	507.14 ± 5.43 b
Yield (kg·ha^-1^)	2582.83 ± 94.92 b	2870.60 ± 171.65 a	2697.36 ± 66.71 ab
Economic output value (¥·ha^-1^)	88022.91 ± 3234.74 b	97830.09 ± 5749.98 a	91925.90 ± 2273.45 ab

The data are mean ± standard deviation (n=3). Different letters in the same row indicated that the parameter was significantly different between different treatments, using one-way analysis of variance, *P* < 0.05.

**Figure 3:**
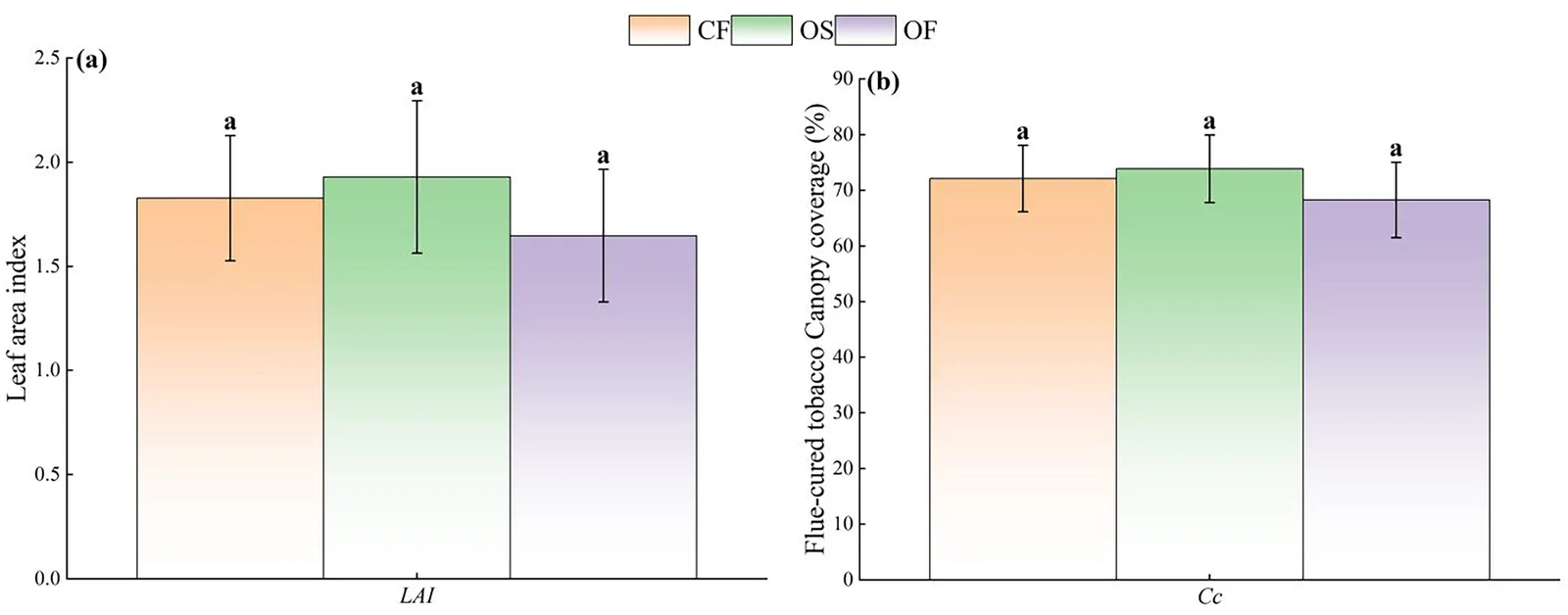
Leaf area index and canopy coverage of flue-cured tobacco under different fertilization regimes. **(a)** Leaf area index of tobacco under different fertilization treatments; **(b)** Canopy coverage of tobacco under different fertilization treatments. All data are mean ± SD, the error bars indicate standard deviation (n=3) and letters express significant differences (*P* < 0.05).

### 3.3 Fertilization regime alters canopy rainfall partitioning across contrasting rainfall events

Fertilization regime significantly affected canopy interception and throughfall (both *P* < 0.001), whereas stemflow was unaffected (*P* = 0.1805). Rainfall event significantly affected all three partitioning components (all *P* < 0.001), with no significant treatment × rainfall event interaction (*P* > 0.05) (**Table 4**). Across the three monitored events, OS consistently showed the highest numerical interception and the lowest numerical throughfall. Interception under OS reached 83.53% during the 22.8-mm event compared with 72.04% under CF. Across treatments, interception fractions declined and throughfall fractions increased from the 22.8-mm to the 85.4-mm event. Because the treatment × rainfall event interaction was not significant, no statistical evidence was obtained that treatment effects differed in magnitude among individual rainfall events.

**Table 4:** Effects of fertilization regime and rainfall event on canopy rainfall partitioning.

Rainfall volume(mm·d^-1^)	Treatments	Canopy interception rate (%)	Stemflow rate (%)	Throughfall rate (%)
22.8 mm	CF	72.04 ± 2.25	13.59 ± 3.08	14.38 ± 0.84
	OS	83.53 ± 3.83	8.94 ± 4.87	7.54 ± 1.11
	OF	76.30 ± 4.25	12.66 ± 3.60	11.04 ± 1.12
42.5 mm	CF	61.28 ± 2.43	6.39 ± 3.07	32.33 ± 0.94
	OS	65.87 ± 0.30	6.29 ± 0.52	27.84 ± 0.78
	OF	63.05 ± 4.14	6.74 ± 3.74	30.21 ± 0.57
85.4 mm	CF	47.94 ± 0.98	6.89 ± 0.53	45.16 ± 1.45
	OS	53.98 ± 0.73	4.77 ± 0.61	41.25 ± 1.24
	OF	51.32 ± 2.83	4.42 ± 1.08	44.27 ± 1.96
Fixed effect P value				
Treatment		**<0.001**	0.1805	**<0.001**
Rainfall event		**<0.001**	**<0.001**	**<0.001**
Treatment × Rainfall event		0.1257	0.3852	0.0979

Data are presented as mean ± SD (n = 3). Treatment, rainfall event, and their interaction were tested using repeated-measures linear mixed-effects models with plot as a random intercept. Because no significant treatment × rainfall event interaction was detected (*P* > 0.05), event-specific significance letters were not assigned. Bold P values indicate significance at *P* < 0.05.

### 3.4 Effects of fertilization regimes on soil aggregate distribution and stability

Fertilization regime altered soil aggregate distribution and stability (**Figure 4**; **Table 5**). OS significantly increased the proportion of large dry-sieved aggregates (>5 mm) and decreased the proportion of microaggregates (<0.25 mm) relative to the other treatments. OS also exhibited the highest WSA_>0.25_, MWD, and GMD values, exceeding CF by 7.20%, 12.61%, and 21.83%, respectively, and OF by 2.96%, 9.28%, and 13.82%. WSA_>0.25_ and GMD differed significantly among all three treatments, whereas MWD was significantly higher under OS than under CF and OF (*P* < 0.05).

**Figure 4:**
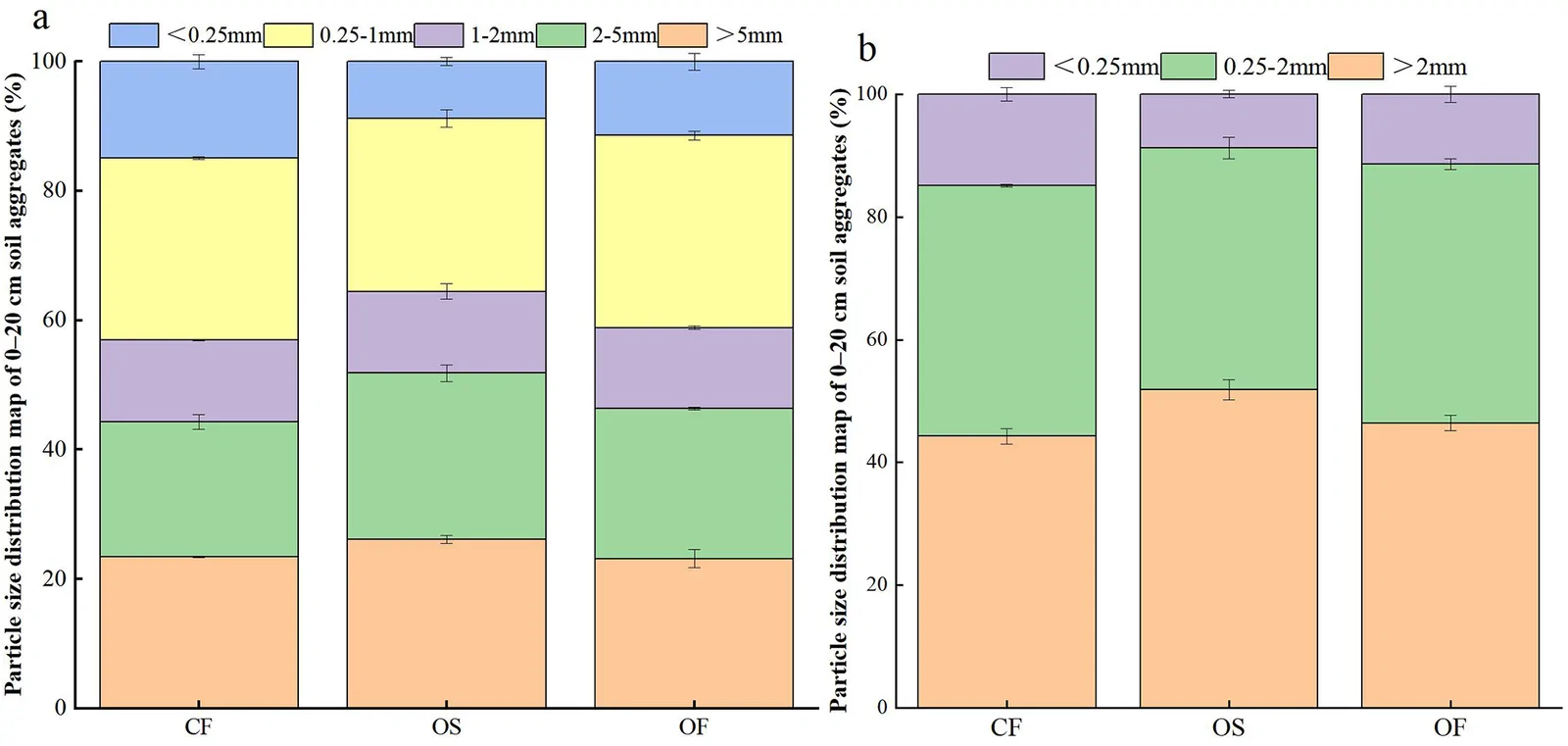
Distribution of soil aggregate size classes under different fertilization treatments. Error bars represent standard deviations of the means (n=3). Panel **(a)** shows the distribution of dry-sieved aggregate fractions (<0.25, 0.25-1, 1-2, 2-5, and >5 mm), while panel **(b)** presents the water-stable aggregate fractions (<0.25, 0.25-2, and >2 mm).

**Table 5:** The influence of different fertilization treatments on soil aggregates.

Treatment	CF	OS	OF
WSA_>0.25_ (%)	85.09 ± 1.08 c	91.22 ± 0.62 a	88.60 ± 1.32 b
MWD	2.30 ± 0.04 b	2.59 ± 0.04 a	2.37 ± 0.06 b
GMD	1.42 ± 0.05 c	1.73 ± 0.04 a	1.52 ± 0.06 b

WSA_>0.25_, proportion of water-stable aggregates >0.25 mm; MWD, mean weight diameter; GMD, geometric mean diameter. Different letters in the same row indicate significant differences between treatments (*P* < 0.05).

### 3.5 Effects of fertilization regimes on runoff and leaching-mediated nutrient export

During the tobacco-growing season, cumulative precipitation reached 827.30 mm, with individual events ranging from 0.30 to 85.40 mm; 53.77% of the total precipitation occurred in August. Seven runoff-producing events generated 34,269.47 L of cumulative runoff across the experimental plots, of which 48.76% occurred in August and 30.17% in September (**Supplementary Figure S1**). Runoff volume increased significantly with rainfall amount (*P* < 0.05). Overall, fertilization regime significantly affected hydrological N and P export, with OS showing the lowest cumulative losses through both surface runoff and subsurface leaching.

#### 3.5.1 Surface runoff nutrient losses

Fertilization regime significantly affected cumulative surface runoff and runoff-mediated nutrient losses (**Table 6**). Relative to CF, OS reduced runoff yield by 7.04% and decreased R-TN, R-NO_3_^--^N, R-TP, and R-TDP losses by 22.46%, 25.97%, 20.63%, and 32.56%, respectively (*P* < 0.05), whereas R-NH_4_^+^-N was unaffected. OF significantly reduced R-TP loss by 17.46% but had no significant effect on runoff yield or the other measured N and P loss components.

**Table 6:** Cumulative runoff volume and nutrient losses during the flue-cured tobacco growing season.

Treatment	Runoff Yield (L)	R-TN (kg·ha^-1^)	R-NH_4_^+^-N (kg·ha^-1^)	R-NO_3_^--^N (kg·ha^-1^)	R-TP (kg·ha^-1^)	R-TDP (kg·ha^-1^)
CF	3940.13 ± 71.93 a	6.99 ± 0.45 a	0.39 ± 0.01 a	3.62 ± 0.25 a	0.63 ± 0.03 a	0.43 ± 0.03 a
OS	3662.79 ± 68.53 b	5.42 ± 0.26 b	0.33 ± 0.05 a	2.68 ± 0.46 b	0.50 ± 0.03 b	0.29 ± 0.04 b
OF	3820.24 ± 175.09 ab	6.48 ± 0.46 a	0.39 ± 0.07 a	3.40 ± 0.26 a	0.52 ± 0.05 b	0.36 ± 0.04 ab

R-TN, means Surface runoff loss of Total Nitrogen; R-NH_4_^+^-N, means Surface runoff loss of Ammonium Nitrogen; R-NO_3_^—^N, means Surface runoff loss of Nitrate Nitrogen; R-TP, means Surface runoff loss of Total Phosphorus; R-TDP, means Surface runoff loss of Total Dissolved Phosphorus. The data are mean ± standard deviation. Different letters in the same column indicated that the parameter was significantly different between different treatments, using one-way analysis of variance, *P* < 0.05.

Across the seven runoff-producing rainfall events, repeated-measures linear mixed-effects models revealed pronounced temporal variation in all measured nutrient-loss components, with a significant effect of runoff event on R-TN, R-NH_4_^+^-N, R-NO_3_^--^N, R-TP, and R-TDP losses (all *P* < 0.001; **Figure 5**). Fertilization treatment significantly affected event-specific R-TN (*P* = 0.009), R-NO_3_^--^N (*P* = 0.007), R-TP (*P* = 0.031), and R-TDP (*P* = 0.006) losses, whereas its effect on R-NH_4_^+^-N was not significant (P = 0.110). The treatment × runoff event interaction was significant only for R-NO_3_^--^N (*P* < 0.001), indicating that treatment effects on nitrate-N export varied among runoff events (**Supplementary Table S3**). Event-specific comparisons showed that OS significantly reduced R-NO_3_^--^N loss relative to both CF and OF during the final two monitored runoff events on 2 and 13 September. In contrast, no significant treatment × runoff event interactions were detected for R-TN (*P* = 0.548), R-NH_4_^+^-N (*P* = 0.300), R-TP (*P* = 0.836), or R-TDP (*P* = 0.598). Across events, OS showed lower estimated marginal mean R-TN and R-TP losses than CF and lower R-TDP loss than both CF and OF.

**Figure 5:**
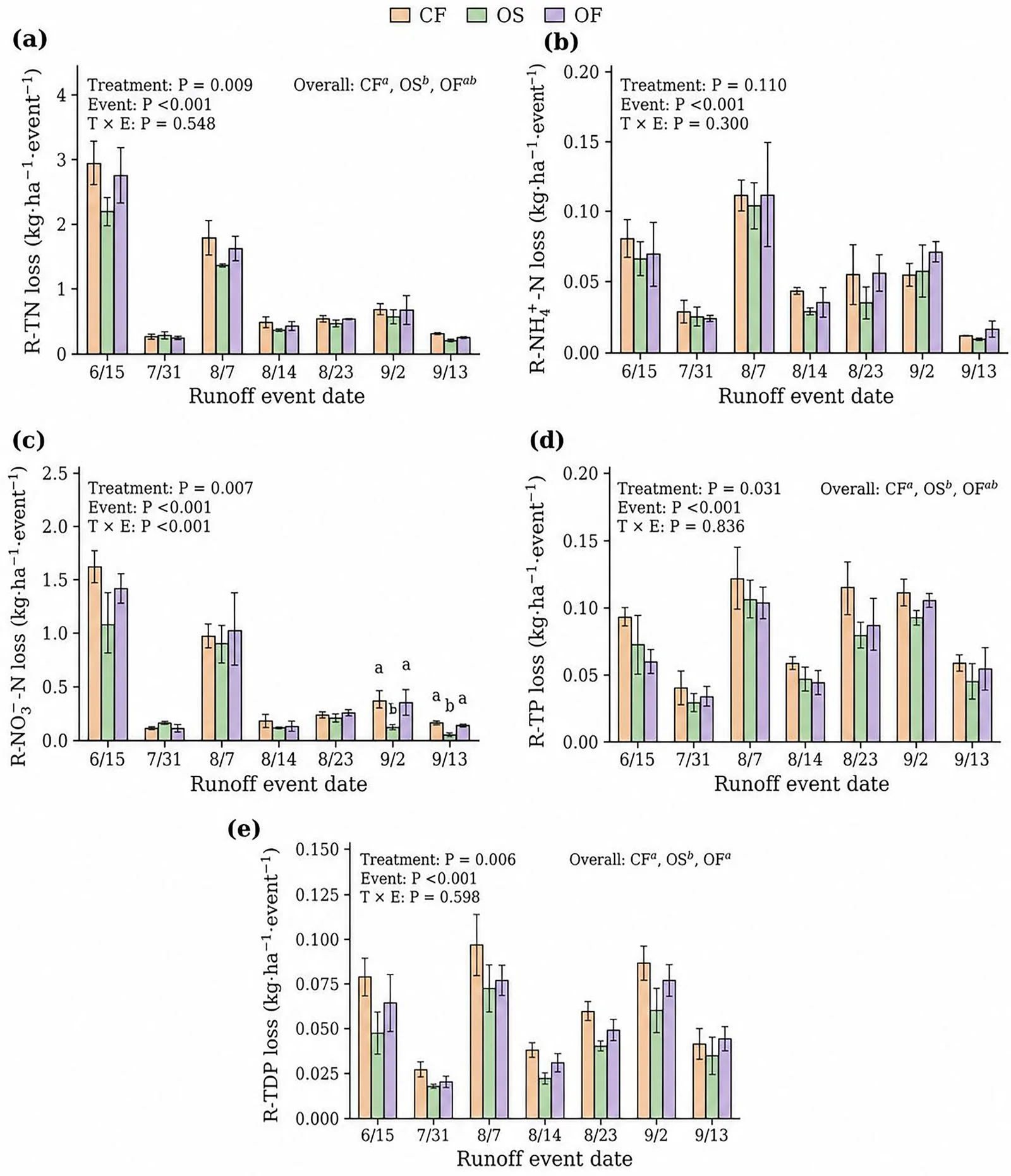
Event-specific runoff-mediated nitrogen and phosphorus losses during the flue-cured tobacco growing season. Panels show losses of **(a)** total nitrogen (R-TN), **(b)** ammonium nitrogen (R-NH_4_^+^-N), **(c)** nitrate nitrogen (R-NO_3_^--^N), **(d)** total phosphorus (R-TP), and **(e)** total dissolved phosphorus (R-TDP) across seven runoff-producing rainfall events. Bars represent untransformed means ± standard deviations of three independent replicate plots per treatment (n = 3). P-values shown in each panel correspond to the fixed effects of treatment, runoff event, and their interaction. For R-NO_3_^−^-N, where the treatment × runoff event interaction was significant, different lowercase letters above bars indicate significant differences among treatments within the same runoff event based on Holm-adjusted pairwise comparisons of estimated marginal means (P < 0.05). For R-TN, R-TP, and R-TDP, where the treatment main effect was significant but the treatment × runoff event interaction was not, overall lowercase-letter groupings indicate Holm-adjusted differences among treatment-level estimated marginal means across runoff events. No treatment-level significance letters are shown for R-NH_4_^+^-N because neither the treatment effect nor the treatment × runoff event interaction was significant.

#### 3.5.2 Nutrient leaching losses

Fertilization regime significantly affected leachate volume and nutrient leaching losses (**Table 7**). Relative to CF, OS reduced leachate amount and L-TN, L-NH_4_^+^-N, L-NO_3_^--^N, and L-TP losses by 10.17%, 24.70%, 25.00%, 26.79%, and 30.61%, respectively (*P* < 0.05). OF significantly reduced L-TN and L-NH_4_^+^-N losses by 14.98% and 18.75%, respectively, but had no significant effect on the other measured leaching components.

**Table 7:** Effects of different treatments on nutrient leaching loss in flue-cured tobacco season (kg·ha^-1^).

Treatment	Leached amount (L)	L-TN (kg·ha^-1^)	L-NH_4_^+^-N (kg·ha^-1^)	L-NO_3_^--^N (kg·ha^-1^)	L-TP (kg·ha^-1^)	L-TDP (kg·ha^-1^)
CF	42.76 ± 0.98 a	4.94 ± 0.27 a	0.32 ± 0.01 a	2.24 ± 0.15 a	0.098 ± 0.009 a	0.007 ± 0.003 a
OS	38.41 ± 0.94 c	3.72 ± 0.33 b	0.24 ± 0.03 b	1.64 ± 0.13 b	0.068 ± 0.007 b	0.005 ± 0.002 a
OF	40.92 ± 0.81 b	4.20 ± 0.40 b	0.26 ± 0.01 b	1.82 ± 0.17 a	0.084 ± 0.015 ab	0.006 ± 0.003 a

L**-**TN, means Leaching loss of Total Nitrogen; L-NH_4_^+^-N, means Leaching loss of Ammonium Nitrogen; L-NO_3_^—^N, means Leaching loss of Nitrate Nitrogen; L-TP, means Leaching loss of Total Phosphorus; L-TDP, means Leaching loss of Total Dissolved Phosphorus. The data are mean ± standard deviation. Different letters in the same column indicated that the parameter was significantly different between different treatments, using one-way analysis of variance, *P* < 0.05.

Leachate concentrations of TN, NH_4_^+^-N, NO_3_^--^N, TP, and TDP generally declined over the tobacco-growing season, with the highest concentrations recorded at the first sampling date (May 30) and lower values toward crop maturity (**Figure 6**). CF generally exhibited the highest nutrient concentrations, whereas OS tended to show the lowest values, particularly for TN, NH_4_^+^-N, NO_3_^--^N, and TP. Treatment differences narrowed during later growth stages and were less pronounced for TDP.

**Figure 6:**
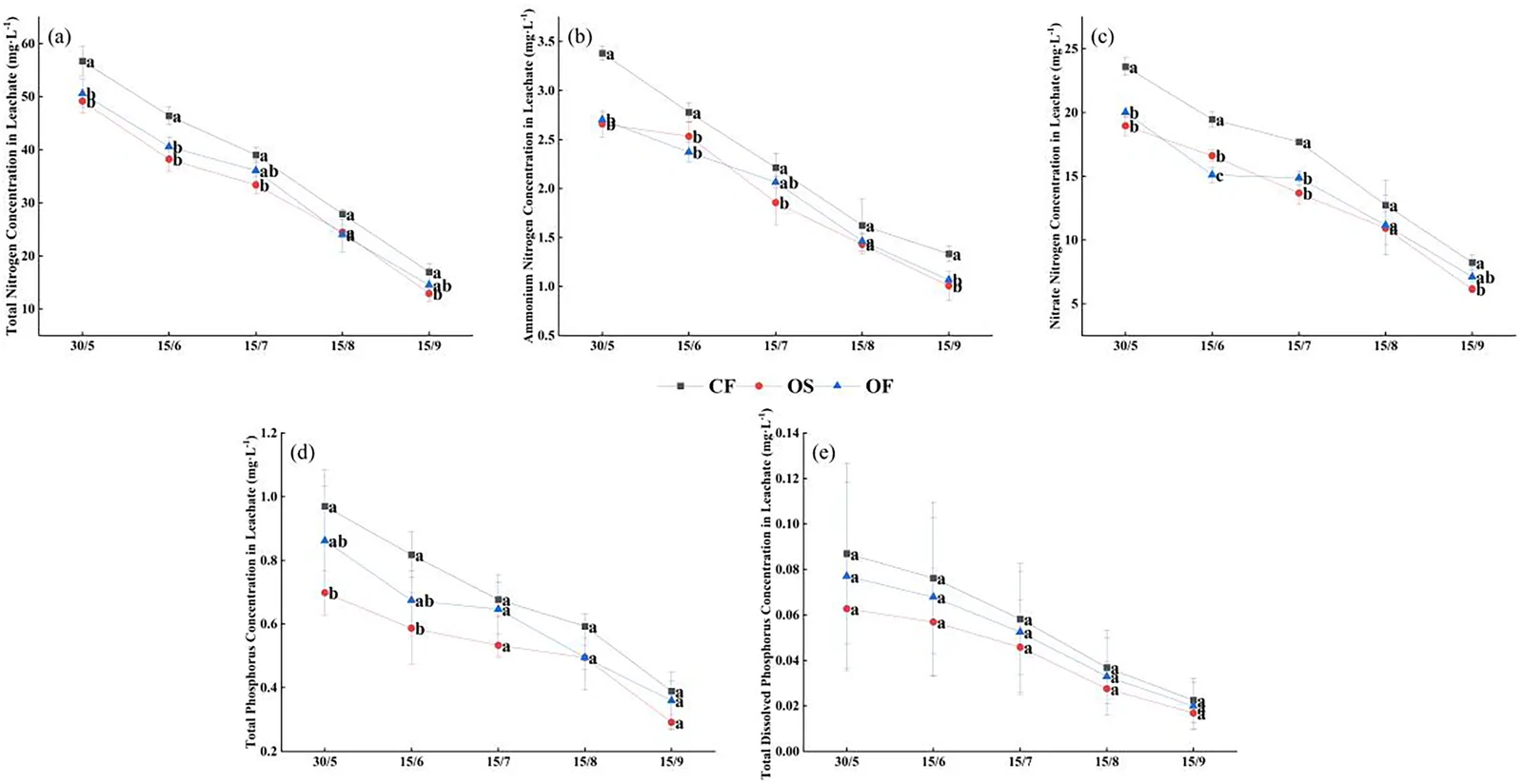
Nutrient concentrations in leachate under different fertilization treatments during tobacco growth. **(a)** Total nitrogen (L-TN); **(b)** Ammonium nitrogen (L-NH_4_^+^-N); **(c)** Nitrate nitrogen (L-NO_3_^--^N); **(d)** Total phosphorus (L-TP); **(e)** Total dissolved phosphorus (L-TDP). Data are presented as mean ± standard deviation (n = 3). Different lowercase letters above the error bars indicate significant differences among treatments at the same sampling time point (p < 0.05).

### 3.6 Associations among canopy interception, soil properties, and nutrient export

Canopy interception rate was negatively associated with nutrient losses through both surface runoff and leaching pathways (**Figures 7a–d**), with stronger relationships observed for surface runoff losses. The relationships between canopy interception and R-TN and R-TP yielded R² values of 0.477 and 0.685, respectively, compared with 0.195 and 0.325 for L-TN and L-TP. These results showed numerically stronger associations between canopy interception and runoff-mediated nutrient export, particularly R-TP, than between canopy interception and leaching-mediated nutrient losses.

**Figure 7:**
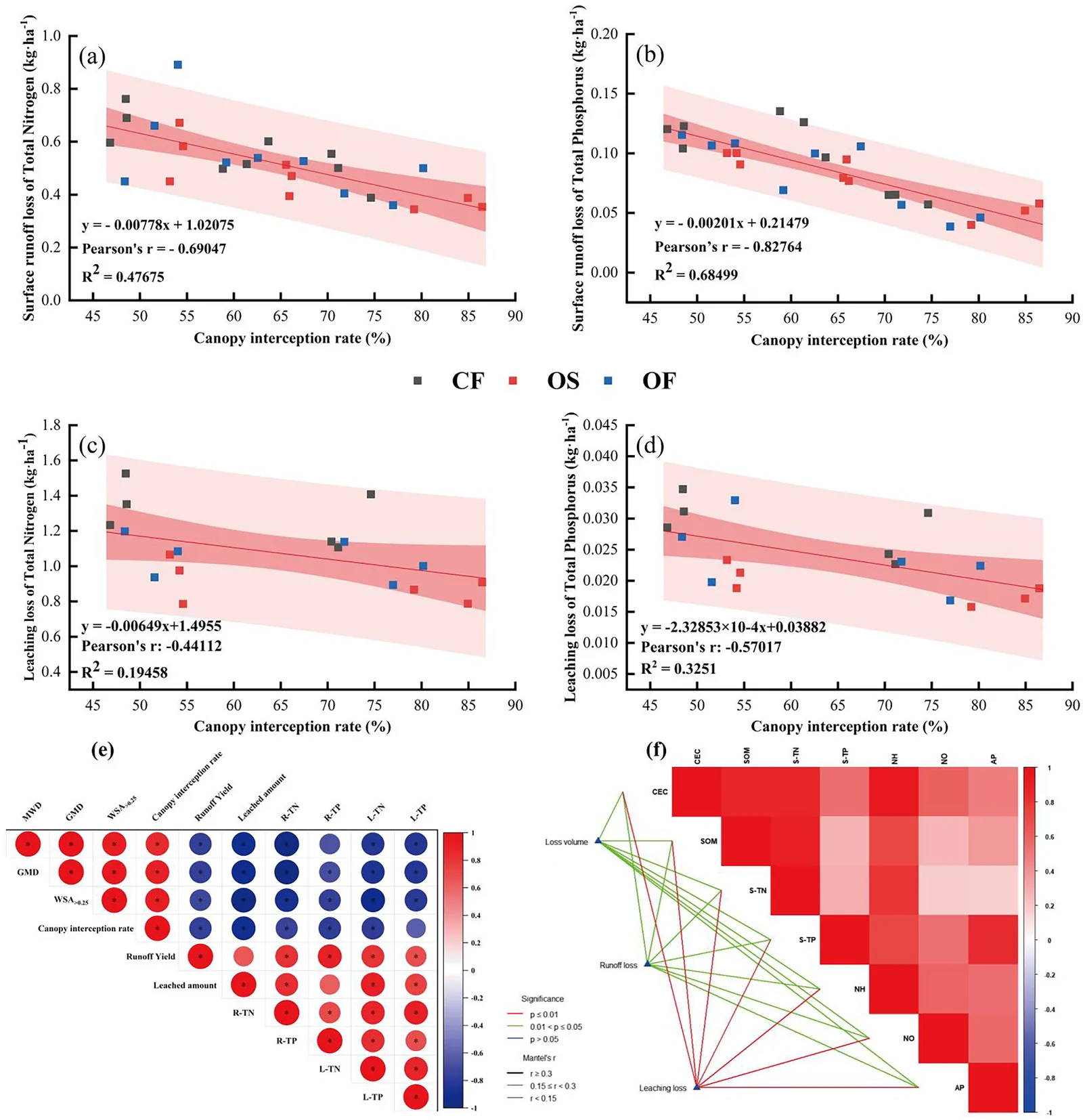
Exploratory associations of canopy interception and soil properties with surface and subsurface nutrient export under different fertilization regimes. **(a–d)** Event-scale relationships between canopy interception rate and runoff- or leaching-mediated TN and TP losses based on 27 plot × rainfall-event observations. Shaded areas indicate 95% confidence intervals. **(e)** Pearson correlations among aggregate stability, canopy interception, hydrological transport, and nutrient-loss variables. Circle color and size indicate correlation direction and magnitude, respectively; asterisks denote *P* < 0.05; **(f)** Mantel associations between soil physicochemical properties and water-loss volume, runoff-mediated nutrient loss, and leaching-mediated nutrient loss. Line width indicates Mantel’s r, and line color denotes significance: red, *P* ≤ 0.01; green, 0.01 < *P* ≤ 0.05; blue, *P* > 0.05.

The Pearson correlation matrix showed close associations among canopy interception, aggregate stability, hydrological fluxes, and nutrient losses (**Figure 7e**). Aggregate stability indicators, including MWD, GMD, and WSA_>0.25_, were positively correlated with canopy interception rate and negatively correlated with runoff yield, leachate volume, and N and P losses. In contrast, runoff yield and leachate volume were positively correlated with their corresponding nutrient loss indicators.

Mantel analysis further showed multivariate associations between soil physicochemical properties and hydrological nutrient-loss pathways (**Figure 7f**). Loss-related variables were grouped into three matrices: loss volume, comprising runoff yield and leachate volume; runoff loss, comprising R-TN and R-TP; and leaching loss, comprising L-TN and L-TP. Soil variables included CEC, SOM, S-TN, S-TP, NH_4_^+^-N, NO_3_^--^N, and AP. Significant Mantel associations were detected between soil properties and loss volume, runoff loss, and leaching loss, indicating coordinated relationships between soil conditions and hydrological nutrient export.

## 4 Discussion

### 4.1 Contrasting fertilization regimes reshape soil resource conditions and organ-specific tobacco growth

Fertilization is a key agricultural management practice that regulates soil nutrient supply, physical structure, and biological activity (Zhang et al., 2023; Wu et al., 2024). Although long-term reliance on chemical fertilizer can rapidly increase nutrient availability, it may also accelerate soil organic C depletion, destabilize aggregate structure, and impair microbial functioning (Xu et al., 2020; Guo et al., 2024). In contrast, organic amendments provide exogenous organic C and promote interactions among mineral particles, organic colloids, roots, and microbial metabolites, thereby improving soil aggregation, pore structure, and water- and nutrient-retention capacity (Cheng et al., 2020; Jiang et al., 2022; Yang et al., 2022; Zhou Z. et al., 2022; Yuan et al., 2023). The magnitude of these effects nevertheless depends on the properties of the organic material, background soil conditions, and duration of application (Chen et al., 2022; Lu et al., 2024; Tang et al., 2024; Liu et al., 2025; Ren et al., 2025; Yang et al., 2025). Our study support this broader understanding but further demonstrate that the three fertilization regimes were associated with distinct soil resource profiles. Compared with CF, OS produced the most comprehensive improvement in soil condition, as indicated by lower bulk density, greater porosity and water content, higher SOM, SOC, DOC, and CEC, and greater proportions of >0.25-mm water-stable aggregates, MWD, and GMD. Increased aggregate stability can enhance resistance to structural disruption while simultaneously modifying pore architecture, water retention, solute transport, and nutrient retention (Li et al., 2020; Chen et al., 2022; Ren et al., 2025). Thus, the effect of OS cannot be interpreted simply as an increase in individual nutrient pools. Rather, OS appears to have strengthened the overall capacity of the soil to retain water and nutrients and buffer short-term fluctuations in resource supply.

These physical improvements were accompanied by enhanced biochemical functioning. Organic inputs provide readily available C substrates for soil microorganisms and can stimulate extracellular enzymes involved in C, N, and P cycling (Liang et al., 2019; Jiang et al., 2022; Yan et al., 2023). Accordingly, the higher DOC concentrations, activities of several soil enzymes, and NH_4_^+^–N concentrations under OS suggest a more active nutrient-transformation environment (Jiang et al., 2022; Li et al., 2024). Collectively, these responses indicate that OS established a coupled soil condition characterized by improved structure, enhanced biochemical turnover, and greater nutrient-retention capacity. Such coupling provides a more stable resource base for subsequent plant nutrient acquisition than changes in nutrient concentration alone. OF exhibited a distinct soil response profile. Compared with CF, OF moderately reduced soil bulk density and increased porosity, CEC, and TK, whereas differences in soil water content, organic C fractions, and most biological indicators were less pronounced than under OS. Its lower AP concentration was consistent with its lower P input. In contrast, OS showed broader concurrent differences across soil physical, chemical, and biochemical properties. Thus, the three treatments are best interpreted as contrasting fertilization regimes with distinct overall soil response profiles: CF represented the conventional nutrient-supply regime, OF showed a comparatively narrower response pattern consistent with its modified nutrient inputs, whereas OS exhibited broader differences in soil resource status and biochemical activity despite receiving an N input comparable to that of CF.

These differences in soil resource status were reflected in tobacco growth, but the responses were strongly organ specific. Previous studies have shown that organic fertilization can enhance dry matter production, leaf physiological activity, yield, and economic return by improving soil conditions and nutrient supply (Zhai et al., 2020; Huang et al., 2024; Zhu et al., 2024; Qi et al., 2025). Consistent with these findings, OS significantly increased the number of effective leaves, maximum leaf width, maximum leaf area, leaf N concentration, plant survival, dry matter production, tobacco yield, and economic output. In contrast, plant height and stem diameter changed little among fertilization regimes. Thus, OS did not stimulate uniform vegetative growth; instead, its benefits were expressed predominantly through leaf formation and expansion and, ultimately, through production of the economic organ. This organ-specific response is physiologically consistent with the source-sink characteristics of flue-cured tobacco. Because leaves constitute the primary harvested organ and apical elongation is restricted after topping, additional resources are more likely to be allocated to the maintenance and expansion of functional leaves than to continued stem elongation (Ceotto and Castelli, 2002; Shi et al., 2025). The higher leaf N concentration under OS may further support this response because N is required for chlorophyll, photosynthetic proteins, and metabolic enzymes and therefore directly contributes to photosynthetic capacity, cell division, and leaf expansion (Evans, 1989; Liu et al., 2018; Mu and Chen, 2021; Sun et al., 2024). The combination of enhanced leaf development and limited changes in plant height and stem diameter therefore appears to reflect crop-specific resource allocation rather than a generalized stimulation of whole-plant vegetative growth.

Importantly, the increase in individual-leaf size under OS did not result in significant differences in *LAI* or *Cc*. Maximum leaf area describes the development of individual leaves, whereas *LAI* and *Cc* integrate leaf number, overlap, orientation, and spatial organization at the canopy scale. The coexistence of larger individual leaves with similar *LAI* and *Cc* therefore indicates that fertilization altered leaf-level development without proportionally increasing total canopy size. This apparent decoupling between leaf-scale morphology and conventional canopy-scale indices provides an important basis for interpreting the contrasting canopy hydrological responses among fertilization regimes.

### 4.2 Canopy rainfall partitioning responses under contrasting fertilization regimes

The crop canopy represents the first biological interface through which precipitation passes before reaching the soil surface. Rainfall is partitioned by the canopy into interception, throughfall, and stemflow, thereby modifying both the quantity and spatial distribution of water entering the soil-plant system (Zheng et al., 2018; Nazari et al., 2020; Zhang et al., 2023; Yao et al., 2025). In the present study, significant treatment effects were detected for canopy interception and throughfall, whereas *LAI* and *Cc* did not differ significantly among fertilization regimes. Across the three monitored rainfall events, OS consistently showed the numerically highest interception and lowest throughfall, whereas OF generally exhibited intermediate numerical responses between OS and CF. During the 22.8-mm rainfall event, for example, interception reached 83.53% under OS compared with 72.04% under CF. Thus, treatment-related variation in rainfall partitioning was not paralleled by corresponding variation in *LAI* or *Cc*. This divergence indicates that conventional canopy-size metrics alone did not fully characterize the canopy hydrological responses observed among fertilization regimes. *LAI* and *Cc* primarily describe foliage quantity and its projected canopy extent, whereas rainfall partitioning reflects the integrated interaction between precipitation and the canopy as a whole. Previous studies have shown that rainfall interception is not only related to the number of leaves, but also associated with other structural characteristics and surface properties (Zheng et al., 2018; Nazari et al., 2020; Murchie and Burgess, 2022; Roth-Nebelsick et al., 2022; Zhang et al., 2023; Yao et al., 2025). The present results are consistent with this general pattern: comparable *LAI* and *Cc* did not necessarily correspond to comparable interception and throughfall. Rather than assigning the observed differences to any single canopy attribute, these findings highlight canopy rainfall partitioning as a complementary hydrological response that captures information not represented by conventional canopy-size metrics. This distinction is particularly relevant when evaluating crop responses to nutrient management, because similar canopy size does not necessarily imply similar redistribution of incoming rainfall.

Rainfall characteristics also contributed substantially to canopy partitioning. As event rainfall increased from 22.8 to 85.4 mm, interception generally declined whereas throughfall increased across treatments. This pattern is consistent with the finite water-storage capacity of plant surfaces: a relatively large fraction of rainfall can be retained by leaves and stems during the early stages of an event, but additional rainfall is increasingly transferred as throughfall and drainage as canopy storage approaches saturation (Lian et al., 2022; Zhang et al., 2023; Liu et al., 2024). Because the monitored events differed in several meteorological and antecedent conditions, the event effect is most appropriately interpreted as an integrated rainfall-event response rather than as an effect of rainfall amount alone. In addition, the fertilization regime × rainfall-event interaction was not significant, indicating no detectable evidence that treatment effects on rainfall partitioning differed among the three monitored events. Overall, canopy rainfall partitioning varied significantly with fertilization regime and rainfall event, whereas LAI and canopy cover showed no significant treatment differences. This mismatch indicates that conventional canopy-size metrics alone do not fully characterize canopy hydrological responses, highlighting rainfall partitioning as a complementary indicator of canopy–rainfall interactions under contrasting fertilization regimes.

### 4.3 Canopy rainfall redistribution and soil structural stability are differentially associated with surface and subsurface nutrient export

Studies of vegetation hydrology and soil erosion have shown that plant canopies influence surface hydrological conditions by intercepting rainfall, redistributing water as throughfall and stemflow, and modifying the kinetic energy of water reaching the soil surface, with important implications for runoff generation and nutrient transport (Liu et al., 2022; Yao et al., 2025). In the present study, OS consistently exhibited greater canopy interception and lower throughfall across the three monitored rainfall events. Relative to CF, OS reduced cumulative surface runoff by 7.04%, while runoff-associated TN and TP losses decreased by 22.46% and 20.63%, respectively. Exploratory correlation analysis further showed that canopy interception was more strongly associated with runoff-mediated nutrient export, particularly runoff TP, than with leaching-mediated nutrient export. These associations were numerically stronger for surface than for subsurface nutrient export, suggesting closer covariation between canopy rainfall redistribution and surface transport under the conditions examined. However, because reductions in N and P export substantially exceeded the decline in runoff volume, variation in nutrient export was not proportional to variation in water flux alone. The observed nutrient loss patterns therefore co-occurred with differences in several other measured components, including mobile nutrient availability, crop nutrient acquisition, and soil nutrient retention properties.

Subsurface nutrient export exhibited a different pattern. Once rainfall infiltrates into soil, downward water and solute movement is closely associated with soil matrix properties, including water-holding capacity, pore connectivity, aggregate stability, and nutrient adsorption–desorption processes (Fukumasu et al., 2024; Rasa et al., 2024). Compared with CF, OS reduced leachate volume by 10.17%, whereas leaching losses of TN and TP declined by 24.70% and 30.61%, respectively. As with surface runoff, the disproportionately larger reductions in nutrient export relative to water flux indicate that reduced drainage volume alone was insufficient to explain the response. The stronger reduction in subsurface nutrient losses under OS was consistent with its improvement in soil structural stability and resource-retention capacity. OS increased the proportion of >0.25 mm water-stable aggregates, MWD, and GMD and was also associated with higher soil water content, organic matter, and CEC. Stable aggregates can increase resistance to dispersion while altering soil pore architecture and water-flow patterns, which may favor greater retention of water and nutrients within the soil matrix (Li et al., 2020; Fukumasu et al., 2024; Rasa et al., 2024). Increased organic matter and CEC further enhance the soil’s capacity to retain nutrient ions. Consequently, whereas surface nutrient export showed closer exploratory covariation with canopy rainfall redistribution, the reduction in subsurface nutrient export under OS appears to have been more strongly associated with the integrated effects of soil structural stability, resource retention, and plant nutrient acquisition.

The contrasting transport behavior of N and P provides further context for interpreting these response patterns. NO_3_^--^N is highly soluble and weakly retained by most soil exchange surfaces and is therefore particularly susceptible to transport with runoff and percolating water. The concurrent increase in plant N status and decrease in NO_3_^--^N export under OS is consistent with altered N allocation within the soil–plant system, although the present measurements do not permit a quantitative N balance interpretation. This interpretation is consistent with previous evidence that organic inputs can stimulate N transformation and plant utilization while reducing the pool of inorganic N vulnerable to transport (Wei et al., 2021; Li et al., 2024). P mobility is governed more strongly by particle detachment, colloidal transport, and adsorption–desorption processes (Liu et al., 2025; Wang et al., 2025). Notably, OS reduced both runoff TP and TDP losses while maintaining higher soil AP at harvest than CF. This coexistence of greater P availability and lower hydrological P export is important because it indicates that improved soil retention did not necessarily occur at the expense of crop-accessible P. Enhanced aggregate stability and organic-matter-associated retention may instead have reduced the movement of particulate, colloidal, and dissolved P while maintaining a biologically available P pool. The coexistence of higher soil AP at harvest and lower hydrological P export indicates that greater residual P availability under OS was not accompanied by greater P loss. OF exhibited a distinct environmental response. Its effects on canopy rainfall redistribution, aggregate stability, and most plant traits were weaker than those observed under OS, but it nevertheless reduced runoff TP and leaching losses of several N forms. Given the lower N and P inputs under OF, these responses are more consistent with nutrient-source control: reducing the amount of nutrient entering the system decreased the quantity potentially available for hydrological export. In contrast, OS achieved nutrient-loss reduction under an N input comparable to CF while simultaneously improving soil structural properties, plant nutrient status, and canopy hydrological functioning.

The three fertilization regimes should therefore be interpreted as exhibiting distinct response profiles rather than representing a simple gradient of treatment effectiveness. CF maintained the conventional nutrient-supply regime and was associated with comparatively greater nutrient export under the conditions of this experiment. OF combined lower N and P inputs with selective reductions in several nutrient-loss components, whereas OS showed broader concurrent differences in soil structural properties, plant nutrient acquisition, canopy rainfall partitioning, and both surface and subsurface nutrient export. These responses occurred simultaneously, and their relative contributions to nutrient-loss reduction cannot be separated by the present experimental design. Accordingly, the principal contribution of this study is the identification of distinct soil–plant–hydrological response profiles among contrasting fertilization regimes, rather than the demonstration of a single causal sequence linking fertilization, canopy development, rainfall redistribution, and nutrient export.

### 4.4 Strengths, limitations, and future perspectives

By integrating fertilization management, soil properties, crop development, canopy rainfall partitioning, and both surface and subsurface nutrient export within the same field experiment, this study provides an integrated field-scale assessment of the responses associated with contrasting fertilization regimes. Rather than indicating a single mode of nutrient-loss mitigation, the results showed distinct response profiles among the three fertilization regimes. OF combined lower N and P inputs with selective reductions in several nutrient-loss components, whereas OS was associated with broader differences in soil resource conditions, plant nutrient acquisition, canopy rainfall partitioning, and hydrological nutrient export. These concurrent responses emphasize the value of considering multiple components of the soil–plant–hydrological system while avoiding attribution of the observed nutrient-loss patterns to any single process.

Several limitations should be acknowledged. The present analysis was restricted to the 2023 tobacco-growing season, so the consistency of these responses across years, sites, soils, and rainfall regimes remains to be tested. In addition, OS and CF received equal N inputs but differed in other nutrients, particularly K. Treatment effects should therefore be attributed to the overall fertilization regimes rather than to N source alone. Canopy rainfall partitioning was monitored during only three late-season rainfall events, whereas runoff and leaching were quantified over longer periods. Thus, the observed relationships indicate coordinated associations rather than a complete causal pathway. Accordingly, the present study was not designed to test statistical mediation or establish a sequential causal pathway from fertilization through plant traits and canopy hydrology to nutrient export. Moreover, several canopy traits were not directly measured, and soil aggregate stability was assessed only at the end of the growing season. These limitations constrain interpretation of the specific processes underlying the observed associations.

Future studies should therefore prioritize multi-season, temporally synchronized measurements and a focused set of variables directly relevant to the hypotheses being tested. Event scale measurements that align rainfall partitioning, runoff, soil water movement, and nutrient concentrations would allow more robust evaluation of their temporal covariation, while targeted measurements of canopy architecture, leaf inclination, canopy porosity, surface wettability, and leaf water-storage properties would help determine why canopies with similar *LAI* differ in rainfall partitioning. Factorial experiments that independently separate fertilizer source, organic C input, and N, P, and K supply would also be required to distinguish the contributions of individual fertilization components. Only with such experimentally discriminating and temporally resolved datasets would formal mediation analysis or structural equation modelling be appropriate for testing proposed causal relationships.

## 5 Conclusion

This study showed that contrasting fertilization regimes were associated with distinct soil, plant, canopy-hydrological, and nutrient-export responses during the 2023 flue-cured tobacco-growing season. Compared with conventional fertilization, organic substitution showed broader concurrent responses, including improved soil resource conditions and aggregate stability, enhanced leaf N status and leaf development, increased crop yield, and lower N and P losses via runoff and leaching. Optimized fertilization produced more moderate soil and plant responses but was accompanied by lower runoff TP and leaching TN and NH_4_^+^-N losses under reduced N and P inputs. Notably, canopy interception and throughfall differed significantly among fertilization regimes despite nonsignificant differences in *LAI* and canopy cover, indicating that conventional canopy-size metrics alone did not fully characterize variation in rainfall partitioning. Exploratory analyses further showed numerically stronger associations between canopy interception and surface nutrient export than with subsurface losses, whereas soil structural properties were more closely associated with subsurface nutrient export. These findings support integrating canopy rainfall partitioning with soil, plant, and nutrient-loss measurements when evaluating fertilization regimes in rainfall-sensitive cropping systems. Multi-season, temporally synchronized experiments are needed to assess the robustness of these associations and further test their potential causal basis.

## Data Availability

The original contributions presented in the study are included in the article/**Supplementary Material**, further inquiries can be directed to the corresponding author/s.
