# Supplementary material for: Canopy rainfall partitioning and hydrological nitrogen and phosphorus losses under contrasting fertilization regimes in a flue-cured tobacco field
Source: Front Plant Sci. 2026 Oct 13;17:1939056. doi: 10.3389/fpls.2026.1939056 (PMC13710098; doi:10.3389/fpls.2026.1939056)
Supplement: Supplementary file 1 [file Supplementaryfile1.docx]

**Canopy rainfall partitioning and hydrological nitrogen and phosphorus losses under contrasting fertilization regimes in a flue-cured tobacco field**

**Yu Chen^1,2^, Wenyin Zi^1,2^, Shan Yang^1^, Yunxue Li^1,2^, Xiangdong Xu^3^, Jing Shi^1,2,*^, Jianjun Chen^1,*^**

1 College of Resources and Environment, Yunnan Agricultural University, Kunming 650201, China

2 Key Laboratory for Improving Quality and Productivity of Arable Land of Yunnan Province, College of Resources and Environment, Yunnan Agricultural University, Kunming, Yunnan 650201, China.

3 College of Agronomy and Horticulture Technology, Yunnan Vocational and Technical College of Agriculture, Kunming 650200, China

* Correspondence: [975407746@qq.com](mailto:975407746@qq.com). Jianjun Chen is the corresponding author, and Jing Shi is a co-corresponding author.

**Table S1** Types and amount of fertilization application of different treatments (kg·ha^-1^)

| Rotation Pattern | Treatments | Base fertilizer | Topdressing | | | Total nutrient input | | |
| --- | --- | --- | --- | --- | --- | --- | --- | --- |
|  |  | Solid organic fertilizer | Liquid organic fertilizer | Potassium sulfate | Compound fertilizer | N | P | K |
| Flue-cured tobacco-Faba bean | CF | 583 | 600 |  | 419.8 | 118.38 | 76.96 | 135.20 |
|  | OS | 3320.83 | 600 |  |  | 118.38 | 79.70 | 228.29 |
|  | OF | 583 | 600 | 63 | 314.85 | 102.64 | 61.22 | 150.96 |

Note: Solid organic fertilizer (N:P₂O₅:K₂O = 2.3:2.4:5.7, %); Liquid organic fertilizer (N:P_2_O_5_:K_2_O=7:0:6.5, %); Potassium sulfate (K₂O=50%); Compound fertilizer (N:P₂O₅:K₂O = 15:15:15, %); “Fold pure total”refers to the calculated total amounts of pure N, P₂O₅, and K₂O applied in each treatment.

**Table S2** Effects of different fertilization treatments on soil organic carbon fractions

| Treatment | CF | OS | OF |
| --- | --- | --- | --- |
| SOM (g·kg^-1^) | 67.74±1.93 b | 74.32±3.01 a | 70.63±3.01ab |
| SOC (g·kg^-1^) | 39.29±1.12 b | 43.11±1.75 a | 40.96±1.75 ab |
| POC (g·kg^-1^) | 12.86±0.56 a | 13.39±0.82 a | 12.99±0.28 a |
| MAOC (g·kg^-1^) | 24.70±0.84 b | 26.64±0.33 a | 25.56±0.88 ab |
| DOC (g·kg^-1^) | 0.86±0.04 b | 1.09±0.05 a | 0.96±0.08 b |
| C/N | 19.47±1.39 a | 17.57±1.39 a | 17.78±0.86 a |

Note: SOM, soil organic matter; SOC, soil organic carbon; POC, particulate organic carbon; MAOC, mineral-associated organic carbon; DOC, dissolved organic carbon; C/N, soil c/n ratio(SOC/TN). Data are presented as mean±standard deviation (n = 3). Different lowercase letters in the same row indicate significant differences among treatments at *p* < 0.05.

**Table S3** Effects of fertilization treatment, runoff event, and their interaction on event-specific runoff-mediated nutrient losses

| Index | Treatment P | Runoff event P | Treatment × Runoff event P |
| --- | --- | --- | --- |
| R-TN | 0.009 | <0.001 | 0.5480 |
| R-NH_4_^+^-N | 0.1096 | <0.001 | 0.2998 |
| R-NO_3_^-^-N | 0.007 | <0.001 | <0.001 |
| R-TP | 0.031 | <0.001 | 0.8356 |
| R-TDP | 0.006 | <0.001 | 0.5979 |

Note: P-values are shown for the fixed effects of fertilization treatment, runoff event, and their interaction based on repeated-measures linear mixed-effects models, with plot identity included as a random intercept. Event-specific nutrient-loss data were natural-log transformed before analysis.

**Fig. S1** Rainfall and runoff yield during flue-cured tobacco season

**Note:** In the figure, daily precipitation is represented by black data points, while vertical bars quantify corresponding surface runoff volumes measured on event days.
